# Prussian Blue Analogues-Derived ZnFe_2_O_4_ in CuO/ZnFe_2_O_4_ p–n Junction for H_2_ Production

**DOI:** 10.1021/acsomega.4c06231

**Published:** 2024-10-16

**Authors:** Linh Trinh, Aleksandra Parzuch, Krzysztof Bienkowski, Piotr Wróbel, Marcin Pisarek, Grzegorz Kaproń, Renata Solarska

**Affiliations:** † Laboratory of Molecular Research for Solar Energy Innovations, Centre of New Technologies, University of Warsaw, Stefana Banacha 2c, 02-097 Warsaw, Poland; ‡ Faculty of Physics, University of Warsaw, Ludwika Pasteura 5, 02-093 Warsaw, Poland; ¶ Institute of Physical Chemistry, Polish Academy of Sciences, Marcina Kasprzaka 44/52, 01-224 Warsaw, Poland; § Faculty of Geology, University of Warsaw, Zwirki i Wigury 93, 02-089 Warsaw, Poland

## Abstract

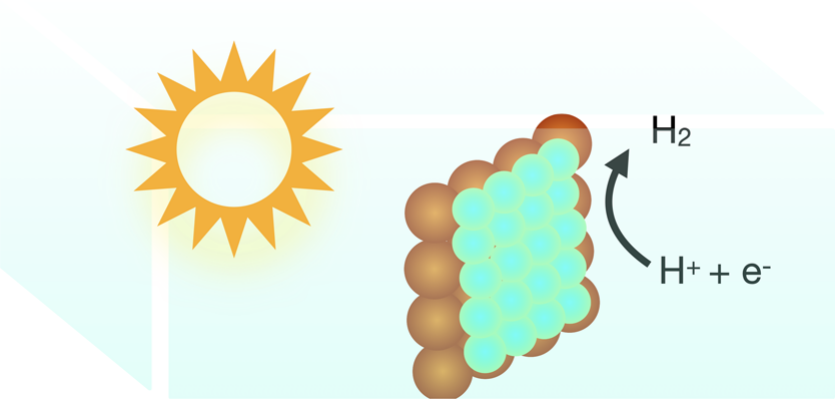

ZnFe_2_O_4_ is an n-type semiconductor spinel oxide with promising applications in various fields, including photocatalysis. This study reports the successful synthesis of a novel ZnFe_2_O_4_ derivative based on Prussian Blue Analogues (PBAs) through an electrosynthesis method. To enhance photocatalytic performance, the synthesized ZnFe_2_O_4_ was employed as a cocatalyst in p–n junction materials, specifically CuO/ZnFe_2_O_4_. Our findings reveal that introducing ZnFe_2_O_4_ could significantly improve the photocatalytic activity and hydrogen (H_2_) production rate of the CuO/ZnFe_2_O_4_ system compared to bare CuO. This enhancement was sustained over an extended operational period, indicating the material’s potential for long-term use in photocatalytic applications. The superior performance of the ZnFe_2_O_4_ cocatalyst is attributed to its efficient charge separation and improved light absorption properties, which collectively contribute to higher photocatalytic efficiency. This study highlights the potential of PBA-based derivative ZnFe_2_O_4_ as an effective cocatalyst in developing advanced photocatalytic systems for sustainable hydrogen production.

## Introduction

High energy consumption and the finite nature of fossil energy reserves drive the rising demand for alternative clean energy sources. Among various approaches, solar-light-driven hydrogen generation using photoelectrochemical (PEC) cells stands out as one of the most attractive and efficient solutions for sustainable energy production.^1,2^ Hydrogen can be obtained from water using inexpensive semiconducting materials and sunlight, making it an ideal candidate for green energy initiatives.

Cupric oxide (CuO), a p-type semiconductor, has garnered significant interest due to its low cost and nontoxicity.^3,4^ Its relatively narrow bandgap of 1.44–1.68 eV further enhances its suitability for solar hydrogen production.^3,5−7^ However, CuO faces challenges related to corrosion, which adversely impacts its stability and photocatalytic performance^8,9^ To address these issues, various strategies have been employed to enhance the stability and photocatalytic efficiency of CuO. Among these strategies, forming heterojunctions by combining semiconductors with different band positions has shown promise in promoting effective electron–hole separation.^10−12^ In particular, p–n junctions have demonstrated superior band gap engineering, significantly enhancing photocatalytic performance.^13−16^ Wang et al. reported a novel strategy to convert p–n junction Cu2O/BiVO4 heterogeneous nanostructures into p–n junction CuO/BiVO_4_ heterogeneous nanostructures via the oxidation of Cu2O to CuO, resulting in CuO/BiVO_4_ heterostructures that exhibited enhanced visible-light-driven photocatalytic activity for degrading rhodamine (RhB) compared to pure BiVO4 nanocrystals, demonstrating the potential of CuO-based p–n junctions to improve photocatalytic performance through effective charge transfer between CuO and BiVO_4_.^17^ Similarly, Meng et al. reported that p–n heterostructures of 2*D*/2D-3D Ni_12_P_5_/ZnIn_2_S_4_ significantly increased photocurrent density and hydrogen evolution rates due to improved charge separation and transportation.^18^

CuO has been incorporated into various systems, such as TiO_2_,^19,20^ ZnO,^13,21−23^ Fe_2_O_3_,^24^ and CdS.^25^ Recently, n-type ZnFe_2_O_4_ has attracted attention due to its suitable band gap (∼2.1 eV) and high theoretical solar-to-hydrogen conversion efficiency (∼20%).^26^ Additionally, ZnFe_2_O_4_ possesses a unique crystal structure, a large specific surface area, and excellent chemical and thermal stability, making it a strong candidate for coupling with CuO to mitigate its limitations.^27^ Despite its potential, the use of ZnFe_2_O_4_ in p–n junctions has not been extensively explored. Notably, a novel p–n heterostructure of n-type ZnFe_2_O_4_ and p-type BiOBr, prepared via an ultrasound deposition method, has shown improved photocatalytic performance.^28^ Additionally, CuO_*x*_-decorated ZnFe_2_O_4_ has demonstrated enhanced photocatalytic and Fenton-like activity due to the p–n junction formed by the two materials.^29^ CuO@ZnFe_2_O_4_ nanoarrays have also exhibited remarkable improvements in photocurrent density.^9^

Recently, our group reported a conventional method of the thermal conversion of metallic hexacyanoferrate (MHCF) or Prussian Blue Analogues to form metal oxides suitable for PEC applications.^30^ In this study, CuOZnFe_2_O_4_ was prepared by sequential electrodeposition and electrosynthesis. The photocathodes underwent surface modification with Zn[Fe(CN)_6_]·xH_2_O (ZnHCF) before being thermally converted into a CuO/ZnFe_2_O_4_ heterostructure. The resulting materials significantly improved the photocurrent density and stability, particularly in hydrogen production. This research underscores the potential of ZnFe_2_O_4_-based p–n junctions for enhancing the performance of PEC cells and advancing the field of solar-driven hydrogen generation.

## Materials and Methods

### Preparation of Electrodes

CuO/ZnFe_2_O_4_ on FTO glass (dimensions of 15 mm × 35 mm) was prepared by electrodeposition using a Biologic SP-300 potentiostat in a standard three-electrode system. This system included an Ag/AgCl reference electrode, a Pt wire as the counter electrode, and clean FTO glass as the working electrode. Initially, the working electrode was held at a constant potential of −0.9 V (vs Ag/AgCl) for 4 min in an 80 mL aqueous solution containing 10 mM CuSO_4_ (CuSO_4_.5H_2_O Sigma-Aldrich 99.99%), 100 mM K_2_SO_4_ (Sigma-Aldrich ≥99.0%), and 1 mM H_2_SO_4_ to deposit Cu. The electrode was then annealed at 550 °C for one h under an O_2_ flow.

Following this, ZnHCF was deposited on the CuO-coated electrode via electrosynthesis using a previously reported method.^31^ The working electrode was cycled between −0.8 and −1.2 V (vs Ag/AgCl) for 10 cycles in an 80 mL aqueous solution containing 0.5 M ZnSO_4_ (ZnSO_4_.7H_2_O, Sigma-Aldrich 99%), 100 mM K_2_SO_4_ (Sigma-Aldrich ≥99.0%), and 10 mM H_2_SO_4_. After deposition, the electrode was soaked in a 20 mL solution of 10 mM K_3_[Fe(CN)_6_] (Sigma-Aldrich ≥99.0%) and 100 mM K_2_SO_4_ for 20 min, then cleaned with water and ethanol, and annealed at 550 °C for 1 h under an O_2_ flow. After annealing, the electrode’s color changed from light yellow to orange-yellow, indicating the formation of ZnFe_2_O_4_. CuO and ZnFe_2_O_4_ films were prepared separately using the same procedures.

### Characterization

The morphology of CuO/ZnFe_2_O_4_ was examined by SEM using a Carl Zeiss Sigma HV workstation, equipped with a Gemini electron column, an energy-selective backscattered detector, and an energy-dispersive X-ray spectrometer (Bruker Quantax XFlash 6—10 detector).

UV–vis spectroscopy was conducted with a Jasco V-650 spectrophotometer equipped with a 60 mm integrating sphere.

Powder X-ray diffraction was performed on samples deposited on FTO glass. Data were collected using an X’Pert PRO MPD powder diffractometer (PANalytical B.V., Netherlands) with Co–Kα radiation (with Fe filter), and the 2θ values were converted to Cu–Kα.

XPS measurements were carried out using a Microlab 350 (Thermo Electron, East Grinstead, UK) spectrometer, with AlKα X-ray radiation at 1486.6 eV. The parameters used were power 300 W, voltage 15 kV, and emission current 20 mA. Elemental XPS spectra were recorded with a pass energy of 40 eV and an energy step size of 0.1 eV. Data were processed using Thermo Fisher’s Advantage software (Version 5.9911) for peak deconvolution, applying an asymmetric Gaussian/Lorentzian mixed function with a constant G/L ratio of 0.35 (±0.05). Binding energies were corrected by using the C 1s peak at 285.0 eV.

### PEC Measurements

PEC measurements were conducted using a three-electrode system with CuO/ZnFe_2_O_4_ on FTO as the working electrode, a platinum wire as the counter electrode, and an Ag/AgCl (saturated KCl) reference electrode in a 0.5 M Na_2_SO_4_ electrolyte (pH 6.5). The electrolyte was purged with argon for 30 min before each measurement. Electrochemical potentials were converted to the RHE scale using the equation: . The working electrodes were polarized at a rate of 10 mV/s by using a CHI660D potentiostat, and simulated AM 1.5G (100 mW cm^2^) illumination was provided by an Oriel 150 W solar simulator.

IPCE vs wavelength data were obtained using a 500 W xenon lamp and a Multispec 257 monochromator (Oriel) with a 4 nm bandwidth. The absolute light intensity was measured with an OL 730–5C UV-enhanced silicon detector (Gooch & Housego). Current vs potential (*J*–*E*) plots for CuO/ZnFe_2_O_4_ photocathodes were measured in a Teflon cell equipped with a quartz window. The exposed surface area of the CuO/ZnFe_2_O_4_ electrode was 0.28 cm^2^.

### PEC H_2_ Production

A Shimadzu Tracera GC-BID gas chromatograph equipped with a PLOT capillary GC, a Zebron ZB-1 capillary column, and GC and BID detectors was used to analyze photocatalytic products. Gaseous samples were tested for possible water reduction products, such as hydrogen, by injecting them into the gas chromatograph using an airtight cylinder. The measurements were conducted using a conventional three-electrode system using a two-compartment Teflon cell with an exposed surface area of 0.5 cm^2^. The system consisted of CuO/ZnFe_2_O_4_ deposited on FTO glass as the working electrode, a platinum wire as the counter electrode, and an Ag/AgCl reference electrode in a 3.5 M KCl solution, all implemented in a 0.5 M Na_2_SO_4_ electrolyte (pH 6.5). A constant potential of −0.6 V vs Ag/AgCl (∼0 V vs RHE) was applied to the electrodes. Simulated AM 1.5G (100 mW/cm^2^) illumination was provided by an Oriel 150 W solar simulator. Gas samples were collected in situ during the photoelectrochemical experiments.

## Results and Discussion

### Morphologies and Structure

**Figure 1:**
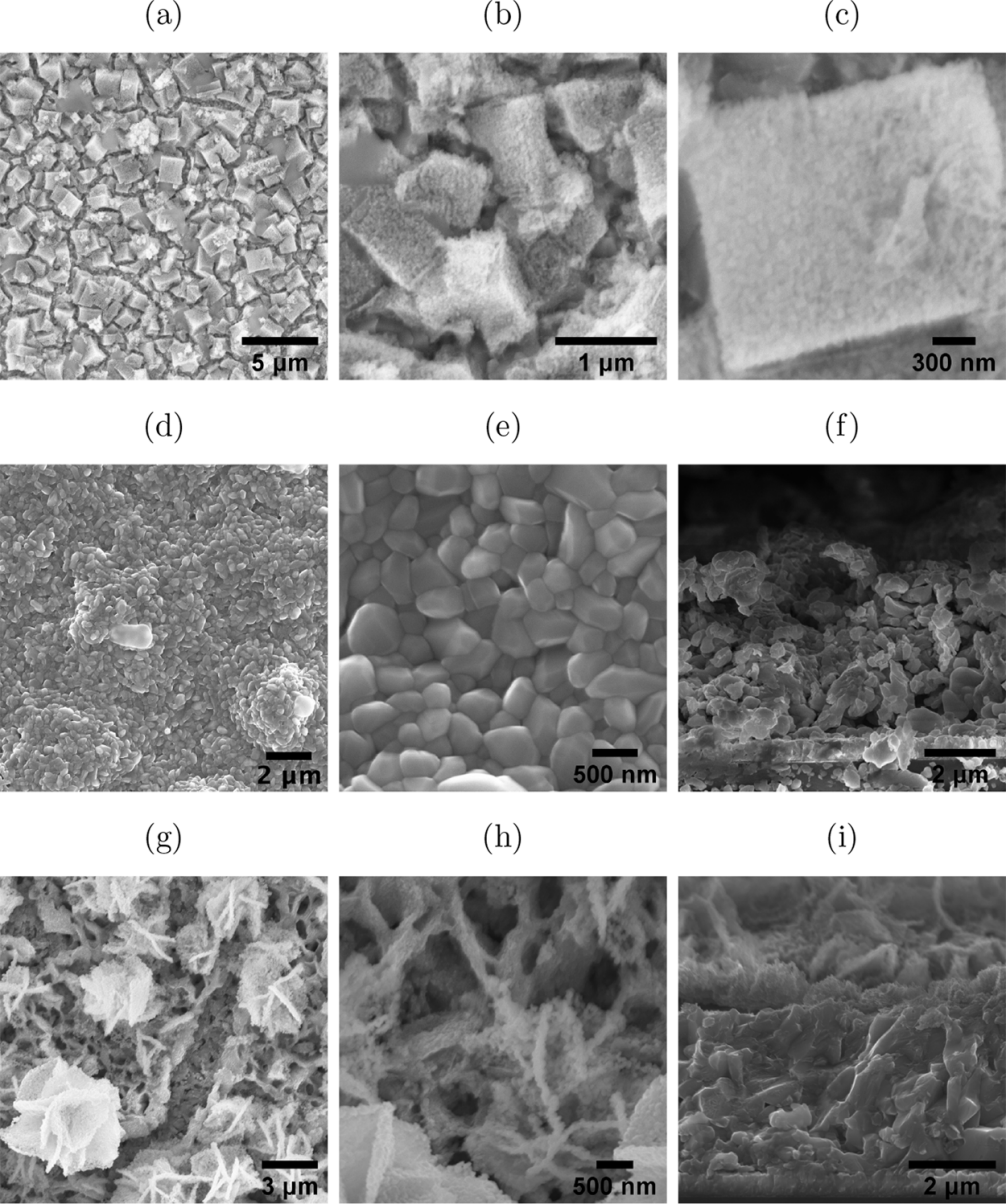
SEM imaging of (a–c) ZnFe_2_O_4_, (d–f) CuO, and (g–i) CuO/ZnFe_2_O_4_.

The morphologies of ZnFe_2_O_4_, CuO, and CuO/ZnFe_2_O_4_ after electrodeposition and heat treatment were characterized by scanning electron microscopy (SEM), as shown in Figure 1. The ZnFe_2_O_4_ layer consists of cubes similar to those of ZnHCF (Figure S1). These cubes contained smaller amorphous particles. The layer is observed to be dense and porous. Energy-dispersive spectroscopy (EDS) analysis showed that the ratio of Zn:Fe:O after thermal treatment is roughly 1:2:4, confirming the formation of ZnFe_2_O_4_ (Figure S2). On the other hand, CuO consisted of large amorphous particles of around 200 nm, making up a porous layer. The energy-dispersive spectroscopy (EDS) analysis showed that the ratio of Cu:O after thermal treatment is roughly 1:1, confirming the formation of CuO (Figure S3). The composition of CuO/ZnFe_2_O_4_ consisted of 1:1 Cu:O and 1:2:4 Zn: Fe:O (Figure S4), confirming the presence of both phases in the structure. Most interestingly, ZnFe_2_O_4_ on CuO appeared in a flower-like structure. The speculation is that, due to the morphologies of the CuO layer, which has small overgrown islands, upon deposition of Zn, Zn^2+^ ions were anchored on these islands, hence the peculiar shapes. These morphologies gave the CuO/ZnFe_2_O_4_ layer a higher surface area and porosity, encouraging better light absorption.

**Figure 2:**
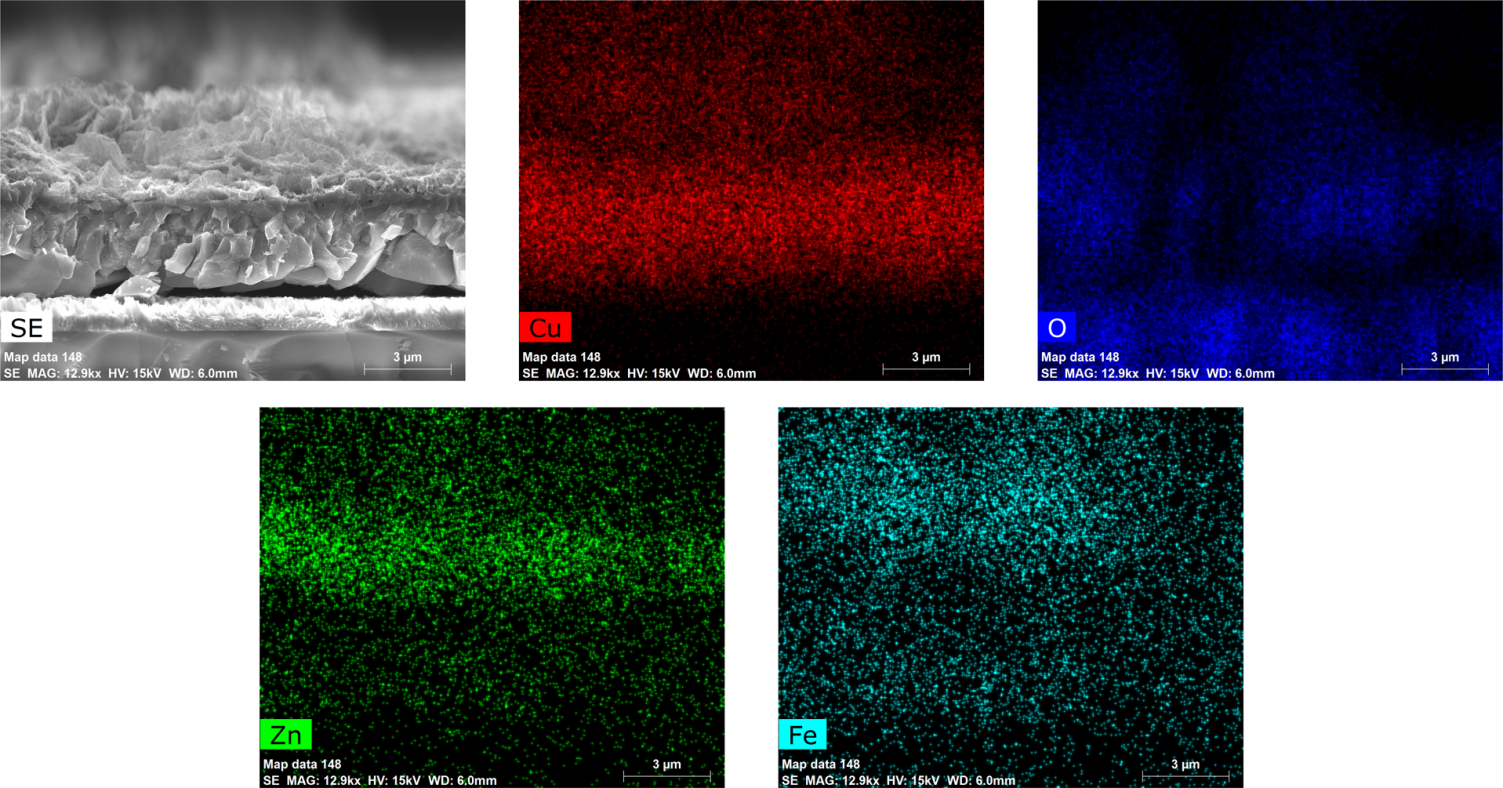
EDS mapping of the cross-section image of CuO/ZnFe_2_O_4_.

EDS mapping of CuO/ZnFe_2_O_4_ at a cross-section shown in Figure 2 again confirmed the structure of the layer, with Cu mainly situated at the bottom layer, while Zn and Fe were mainly situated on the top layer. These results confirmed that the film indeed consisted of two layers that are well in contact with one another.

**Figure 3:**
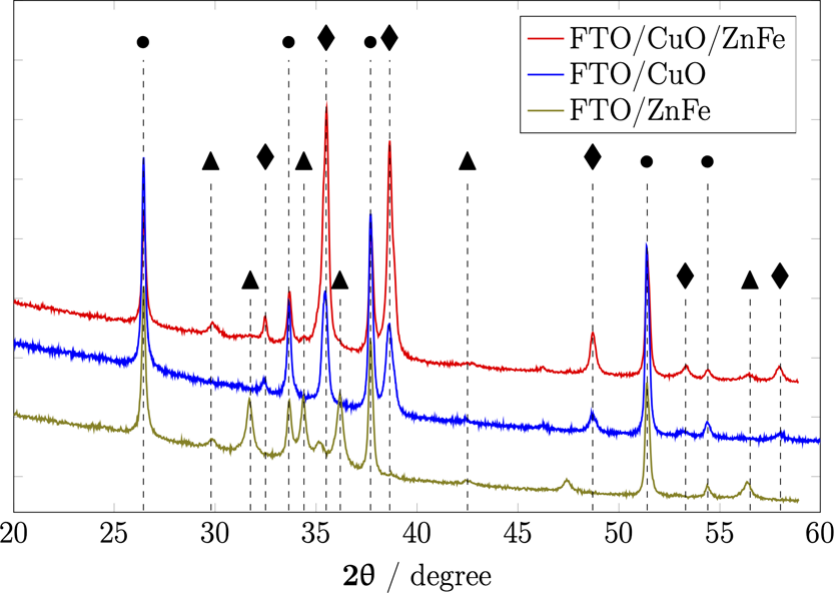
XRD patterns of CuO, CuO/ZnFe_2_O_4_, and ZnFe_2_O_4_ (peak assignment: • = FTO, ▲ = ZnFe_2_O_4_, ⧫ = CuO).

XRD patterns of CuO/ZnFe_2_O_4_, CuO, and ZnFe_2_O_4_ are presented in Figure 3. All the peaks on the XRD pattern of CuO/ZnFe_2_O_4_ reconciled with the peaks of CuO and ZnFe_2_O_4_ in their respective XRD patterns, which confirmed the existence of both phases on the CuO/ZnFe_2_O_4_ electrode (Figure 3). The peaks of ZnFe_2_O_4_ on both ZnFe_2_O_4_ and CuO/ZnFe_2_O_4_ can be indexed at 29.8, 34.4, 36.2, 42.5, and 56.5° as (220), (311), (400), (422), and (440), respectively, of cubic ZnFe_2_O_4_ (JCPDS 22-1012 - Figure S5), confirming its spinel cubic structure.^32^ The peaks of CuO of both CuO/ZnFe_2_O_4_ and CuO, on the other hand, can be indexed at 32.5, 53.5, 38.65, 48.7, 53.5, and 58.0° as (110), (002), (111), (−202), (020), and (202), respectively, of monoclinic CuO (JCPDS-48-1548 - Figure S5).^33^ The grain size of the samples can be obtained by applying the Scherrer equation:
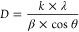
1where *k* = 0.94, λ = 1.54 Å, and β is full-width half-maximum in radian. The sizes of CuO for CuO and CuO/ZnFe_2_O_4_ are around 28.5 nm, which greatly agrees with the grain size observed on SEM, indicating the monocrystallization of CuO in both samples. The sizes of ZnFe_2_O_4_ for ZnFe_2_O_4_ and CuO/ZnFe_2_O_4_ are around 9 nm, which also agrees with what was observed on SEM. The lattice constant *a* of ZnFe_2_O_4_ can be calculated using the following relation:
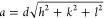
2where *d* is the interplanar distance, which can be calculated by
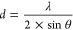
3and *h*, *k*, and *l* are the Miller indices. The *a* value was estimated to be around 9.3 Å, which is higher than the previously reported value for conventional ZnFe_2_O_4_, which is around 8.4 Å.^34,35^ The increase of the lattice constant has also been observed on CuFe_2_O_4_ converted from CuHCF.^30^ This phenomenon has been suggested to be attributed to the decrease in the sizes of nanomaterials.^36^ However, in this case, it can also be attributed to the initial structure of the ZnHCF precursors, which have a lattice constant of around 10 Å.

**Figure 4:**
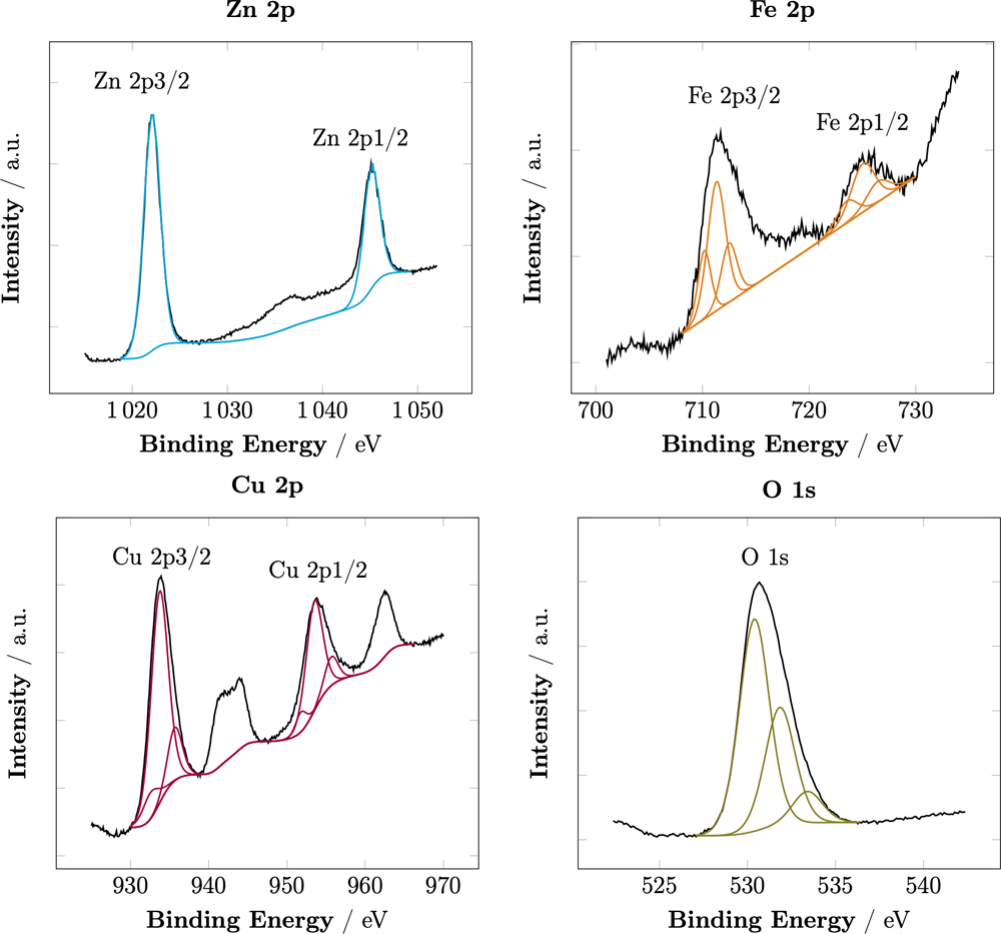
High-resolution deconvoluted XPS spectra of Zn 2p, Fe 2p, Cu 2p, and O 1s of CuO/ZnFe_2_O_4_.

Figure 4 shows the high-resolution XPS peaks of Zn, Fe, Cu, and O and their deconvolutions. The characteristic peaks of Zn 2p_3/2_ at 1021.7 and Zn 2p_1/2_ at 1044.7 demonstrate the existence of Zn^2+^.^9^ The peaks of Fe 2p at 710.9 and 724.7 correspond to Fe 2p3/2 and Fe 2p1/2, respectively, indicating the electronic state of Fe^3+^ in ZnFe_2_O_4_.^37,38^ We can also observe that the Fe^3+^ signal breaks down into two components, 710.9 and 712.1 eV, which can be attributed to octahedral and tetrahedral Fe^3+^, respectively.^38,39^ Moreover, after deconvolution, an additional signal can be distinguished, characteristic of Fe^2+^.^40^ In the Cu 2p XPS spectrum, the strongest peaks at 933.3 and 953.1 indicate the oxidation state II of Cu from CuO.^38,41^ There are also much weaker signals which can be assigned to Cu^1+^ and Cu^2+^ in hydroxides.^42^ For all metallic elements, characteristic satellite lines are visible, the location of which suggests the presence of Fe_3+_, Cu^2+^, and Zn^2+^ ions in the structure of the investigated material.^40,42^ Deconvolution of O 1s shows peaks at 529.6 and 530.9, which belong to the lattice oxygen bound to metal oxides; the peak at 533.5 could be the result of the adsorbed oxygen on the surface such as C–O and C=O functional groups.^43,44^ Overall, the XPS results indeed suggested the formation of CuO/ZnFe_2_O_4_.^44,45^

### Optical Studies

**Figure 5:**
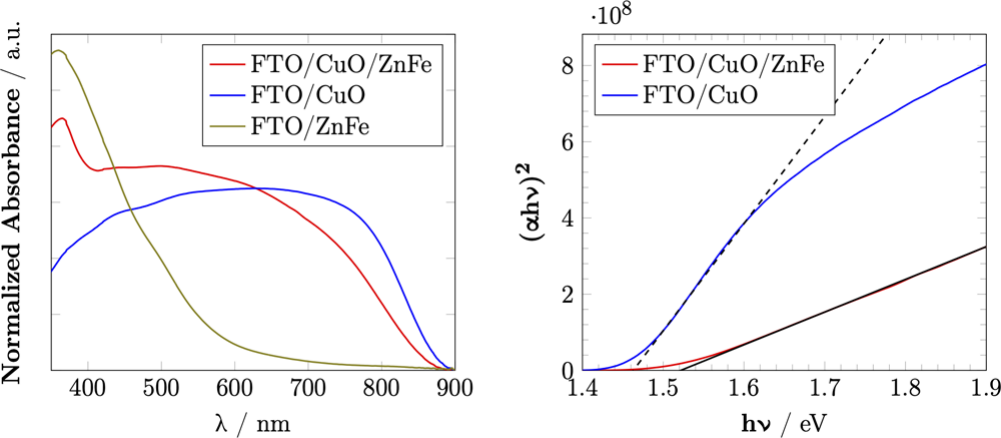
UV–vis spectra of CuO/ZnFe_2_O_4_, CuO, and ZnFe_2_O_4_ after annealing (left) and Tauc plot of CuO/ZnFe_2_O_4_ and CuO (right).

The UV–vis spectra of CuO/ZnFe_2_O_4_, CuO, and ZnFe_2_O_4_ are presented in Figure 5-left. The spectrum of CuO/ZnFe_2_O_4_ shows a strong absorption band in the 500–800 nm range, similar to CuO’s absorption characteristics. However, a notable difference is observed in the absorption band around 370 nm, which can be attributed to the presence of ZnFe_2_O_4_. Furthermore, an increase in the absorption band is observed from 350 to 600 nm, indicating enhanced light harvesting in the visible range due to the incorporation of ZnFe_2_O_4_.

The Tauc plots for the direct band gaps of CuO/ZnFe_2_O_4_ and CuO are shown in Figure 5-right. The CuO sample exhibited a direct band gap of 1.46 eV, whereas the CuO/ZnFe_2_O_4_ composite demonstrated a slightly increased direct band gap of 1.52 eV. In contrast, ZnFe_2_O_4_ exhibited an indirect band gap of 2.19 eV (Figure S6), consistent with previously reported values.^46,47^

The observed band gap values suggest that the CuO/ZnFe_2_O_4_ composite is well-suited for absorbing visible light, making it a strong candidate for solar-driven photocatalysis. The combination of CuO and ZnFe_2_O_4_ in the composite material significantly broadens the absorption spectrum and enhances the efficiency of light harvesting. This enhancement is primarily attributed to the formation of a p–n junction between CuO and ZnFe_2_O_4_. This junction is crucial in facilitating better charge separation and reducing the recombination rate of photogenerated electron–hole pairs.

### Photoelectrochemical Properties

**Figure 6:**
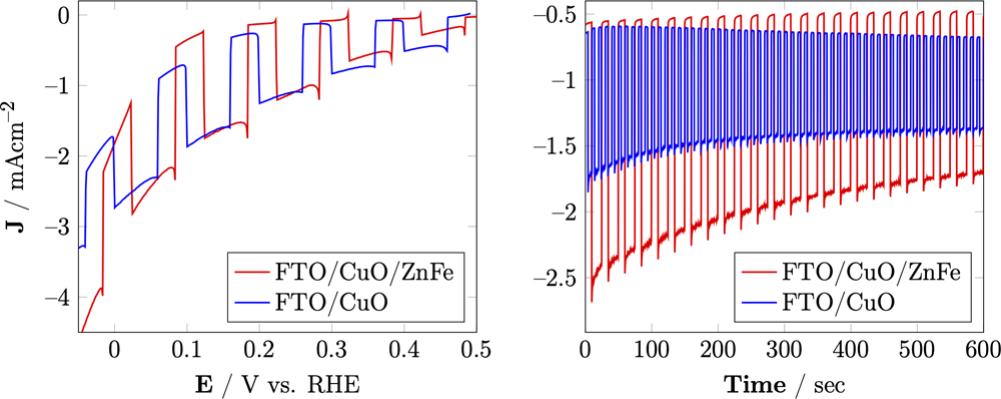
LSV (left) and amperometry (right) of CuO/ZnFe_2_O_4_ and CuO at 0.1 V vs RHE in 0.5 M Na_2_SO_4_ (pH = 6.5).

CuO/ZnFe_2_O_4_ and CuO were evaluated for their photocatalytic properties by using linear sweep voltammetry (LSV). The cathodic scan of the two samples is presented in Figure 6-left. Both electrodes exhibited evident cathodic characteristics typical of p-type materials. CuO displayed a maximum photocurrent density of 1.5 mA/cm^2^ at 0.1 V vs RHE. However, the photocurrent density of CuO significantly decreased below 0 V vs RHE.

In contrast, the CuO/ZnFe_2_O_4_ composite showed a higher maximum photocurrent density of approximately 2 mA/cm^2^ at 0.1 V vs RHE. Notably, unlike CuO, the CuO/ZnFe_2_O_4_ composite maintained this maximum photocurrent density even at 0 V vs RHE, indicating its superior potential for hydrogen (H_2_) production. This result suggests that incorporating ZnFe_2_O_4_ into the CuO matrix enhances the overall photocatalytic performance, likely due to improved charge separation and reduced recombination of electron–hole pairs facilitated by the p–n junction.

ZnFe_2_O_4_ alone displayed a typical n-type anodic current with a maximum photocurrent density of 6 μA/cm^2^ at 1.3 V vs RHE (Figure S7), underscoring the complementary roles of CuO and ZnFe_2_O_4_ in the composite material. This n-type behavior enhances the overall photocatalytic properties of the CuO/ZnFe_2_O_4_ system, as the combination extends the photoresponse range and improves the system’s efficiency.

Stability studies of CuO/ZnFe_2_O_4_ and CuO were conducted using the chronoamperometry technique at 0 V vs RHE, as shown in Figure 6-right. The CuO/ZnFe_2_O_4_ composite exhibited an initial photocurrent density of −2.1 mA/cm^2^, while CuO showed a lower initial photocurrent density of −1.3 mA/cm^2^. Over 10 min of chopped light irradiation, CuO/ZnFe_2_O_4_ retained approximately 70% of its initial photocurrent density, whereas CuO retained only about 53%. This significant difference indicates that the CuO/ZnFe_2_O_4_ composite not only enhances light absorption but also improves the photocatalytic system’s stability.

The improved performance of CuO/ZnFe_2_O_4_ can be attributed to the efficient charge separation and extended light absorption, facilitated by the ZnFe_2_O_4_ component. The formation of a p–n junction between CuO and ZnFe_2_O_4_ is likely responsible for the enhanced stability and photocurrent density, making this composite a promising candidate for solar-driven hydrogen production applications. Furthermore, the unique morphology of the CuO/ZnFe_2_O_4_ composite, with its higher surface area and porosity, provides better light absorption and stability under illumination, addressing a critical issue in photocatalysis.

**Figure 7:**
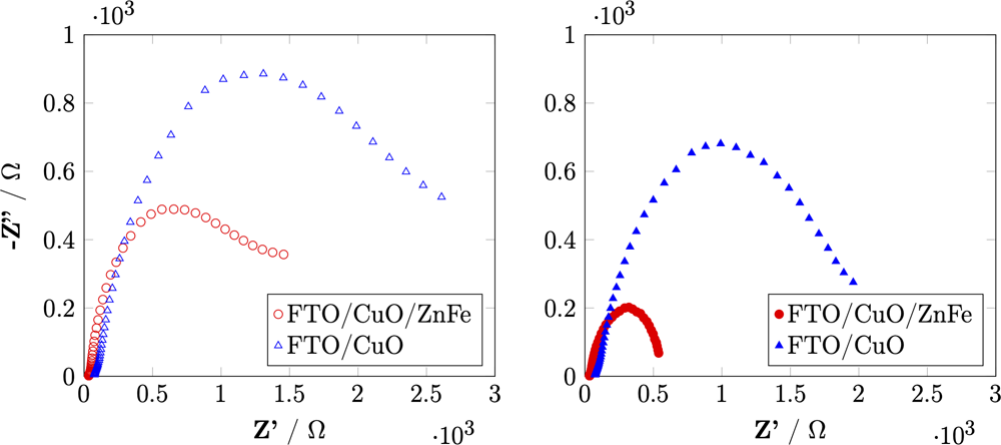
Nyquist plot of CuO/ZnFe_2_O_4_ and CuO under dark and light conditions at 0 V RHE.

Further analysis using the impedance Nyquist plot in Figure 7 reveals a single semicircle for both the CuO/ZnFe_2_O_4_ and CuO samples, indicating that the charge transfer process occurs between the solid phase and the electrolyte. The formation of a p–n junction between CuO (p-type) and ZnFe_2_O_4_ (n-type) enhances the separation of photogenerated electron–hole pairs, reducing the level of charge recombination. The negligible charge transfer resistance between the CuO and ZnFe_2_O_4_ phases confirms the good adhesion of the ZnFe_2_O_4_ structure on CuO. This result ensures efficient charge transport without significant losses due to charge recombination or accumulation at the surface, reducing the extent of photo corrosion in CuO. Additionally, the CuO/ZnFe_2_O_4_ electrode exhibits a smaller Nyquist plot radius compared to CuO, signifying a lower charge transfer resistance (*R*_ct_). The p–n junction formed by the integration of ZnFe_2_O_4_ further enhances the separation and transport of photogenerated charge carriers, contributing to the collection of photogenerated electrons on the CuO surface.

**Figure 8:**
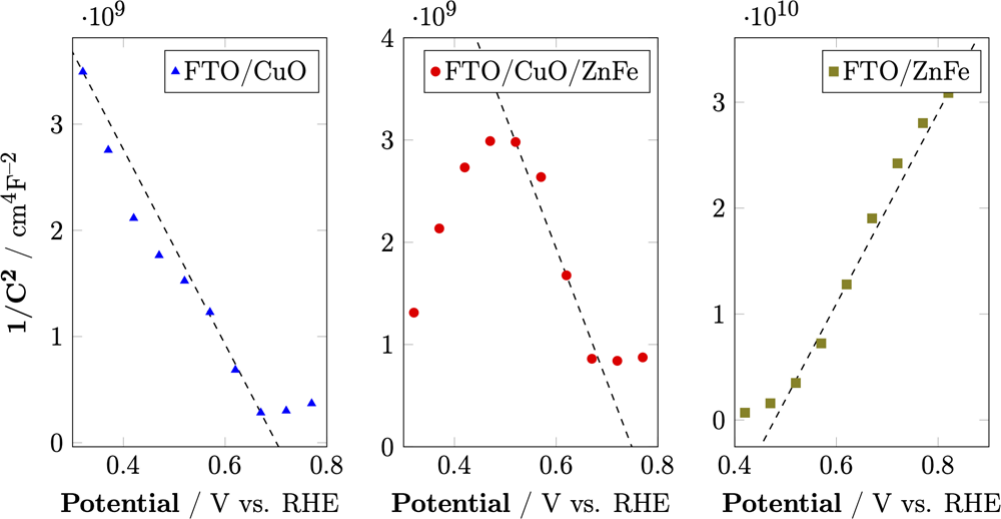
Mott–Schottky plot of CuO, CuO/ZnFe_2_O_4_, and ZnFe_2_O_4_ at 1 kHz.

Mott–Schottky analysis has also been performed to determine the flat band potential (*E*_fb_) of CuO/ZnFe_2_O_4_, CuO, and ZnFe_2_O_4_, as shown in Figure 8. CuO/ZnFe_2_O_4_ and CuO both showed negative slopes, characteristic of p-type semiconductors, while ZnFe_2_O_4_ showed a positive slope, characteristic of n-type materials. The Mott–Schottky plot is described as follows:
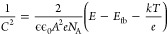
4where *C* is the space-charge capacitance (F^–2^cm^4^), ϵ is the relative permittivity of the semiconductor (with ϵ_CuO_ = 10.26^48^ and ϵ_ZnFe_2_O_4__ = 100),^49^ ϵ_0_ is the permittivity of the free space (8.85 × 10^–12^ Fm^–1^), *e* is the electron charge (1.6 × 10^–19^ C), *A* is the surface area (2 cm^2^), *N*_A_ is the free carrier density, and *k* is the Boltzmann constant (1.38 × 10^–23^ JK^–1^). *T* is the temperature (296 K).^50^ The obtained *N*_A_ values of CuO and ZnFe_2_O_4_ are 1.67 × 10^20^ cm^–3^ 1.09 × 10^19^ cm^–3^, respectively. From there, the flat band potentials of CuO and ZnFe_2_O_4_ can be determined as 0.6 and 0.45 V, respectively.

**Figure 9:**
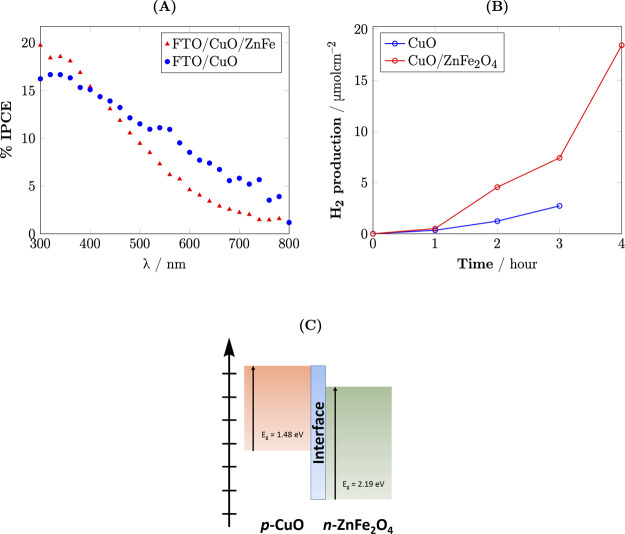
(A) %IPCE of CuO/ZnFe_2_O_4_ and CuO in Na_2_SO_4_ 0.5 M at 0 V vs RHE; (B) H_2_ production at 0 V vs RHE of CuO and CuO/ZnFe_2_O_4_; and (C) scheme of CuO/ZnFe_2_O_4_.

The IPCE measurement was conducted at 0.2 V vs RHE to characterize the photoactivity of CuO/ZnFe_2_O_4_ and CuO at various wavelengths (Figure 9A). The IPCE (%) can be calculated as follows:
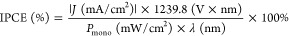
5where *J* is the photocurrent density, *P*_mono_ is the intensity of the monochromatic light recorded with a power meter equipped with a thermopile detector and a calibrated silicon photodiode, and λ is the wavelength of the incident light.

The results indicated that CuO/ZnFe_2_O_4_ exhibited a maximum %IPCE value of 20% at approximately 300 nm, whereas CuO displayed a maximum IPCE value of around 16% at approximately 330 nm. The higher IPCE value observed for CuO/ZnFe_2_O_4_ in the near-UV region is attributed to the presence of ZnFe_2_O_4_, as confirmed by UV–vis spectroscopy. The higher IPCE values and improved light absorption capabilities of CuO/ZnFe_2_O_4_ demonstrate its superior photoactivity compared to pure CuO, which, in turn, makes the composite material a promising candidate for applications in photoelectrochemical cells and solar-driven hydrogen production, where efficient utilization of incident light is crucial. Combining CuO and ZnFe_2_O_4_ extends the light absorption range and enhances the photocatalytic efficiency through improved charge separation and stability.

CuO and CuO/ZnFe_2_O_4_ were applied a biased potential at 0 V vs RHE in a two-compartment cell to investigate the materials’ PEC H_2_ generation. The obtained gases in the chamber of the photoelectrodes were analyzed by gas chromatography, as shown in Figure 9B.

The amount of H_2_ gas that evolved after 2 h on CuO/ZnFe_2_O_4_ was more than double that of CuO, confirming the photocatalytic enhancement. More importantly, the H_2_ generation of CuO/ZnFe_2_O_4_ persistently increased up to 4 h of irradiation, indicating its long-term stability in the hydrogen evolution reaction.

The band positions of CuO and ZnFe_2_O_4_ were calculated from the flat band potential (*E*_fb_) and carrier density (*N*_A_), as determined from Mott–Schottky analysis, along with the band gap (*E*_g_) obtained from UV–vis spectroscopy. The valence band (*E*_vb_) and conduction band (*E*_cb_) positions are shown schematically in Figure 9C, illustrating the favorable band alignment that facilitates efficient charge separation in the CuO/ZnFe_2_O_4_ composite.

## Conclusions

In conclusion, our novel two-step electrosynthesis method has successfully prepared the CuO/ZnFe_2_O_4_ composite. The resultant materials feature a distinctive morphology characterized by flower-like ZnFe_2_O_4_ structures on the CuO layer. This unique morphology significantly enhances the light-harvesting capability of the composite material, leading to improved photocatalytic activity and increased H_2_ gas generation compared to bare CuO.

The enhanced photocatalytic performance of CuO/ZnFe_2_O_4_ can be attributed to several factors. First, forming a p–n junction between p-type CuO and n-type ZnFe_2_O_4_ facilitates efficient charge separation and reduces electron–hole recombination, thus improving photocatalytic efficiency. Second, the flower-like ZnFe_2_O_4_ structures provide a larger surface area and higher porosity, contributing to better light absorption and increased active sites for photocatalytic reactions.

Furthermore, the stability tests conducted using chronoamperometry indicate that the CuO/ZnFe_2_O_4_ composite retains a higher percentage of its initial photocurrent density over time than that of pure CuO, highlighting its enhanced stability under operational conditions. The incident photon-to-current efficiency (%IPCE) measurements further confirm that CuO/ZnFe_2_O_4_ exhibits superior photoactivity across a range of wavelengths, particularly in the near-UV region, due to the presence of ZnFe_2_O_4_.

Overall, the CuO/ZnFe_2_O_4_ composite significantly improves photocatalytic activity and stability, making it a highly effective material for photocatalytic applications under visible light irradiation. These findings suggest that the CuO/ZnFe_2_O_4_ composite holds excellent promise for solar-driven hydrogen production and other photoelectrochemical applications, offering a viable and efficient solution for renewable energy generation.

## Supporting Information Available

Additional data and analyses; SEM images of ZnHCF before annealing (Figure S1); EDS analysis of ZnFe_2_O_4_ after annealing (Figure S2); EDS analysis of CuO (Figure S3) and CuO/ZnFe_2_O_4_(Figure S4); XRD patterns of standard CuO, ZnFe_2_O_4_, and FTO (Figure S5); UV–vis spectra and Tauc plot of ZnFe_2_O_4_(Figure S6); and LSV of ZnFe_2_O_4_ in 0.5 M Na_2_SO_4_ (Figure S7) (PDF)
