# Supplementary material for: Prussian Blue Analogues-Derived ZnFe2O4 in CuO/ZnFe2O4 p–n Junction for H2 Production
Source: ACS Omega. 2024 Oct 16;9(43):43734–42. doi: 10.1021/acsomega.4c06231 (PMC11525516; doi:10.1021/acsomega.4c06231)
Supplement: Supplementary file 1 — ao4c06231_si_001.pdf [file ao4c06231_si_001.pdf]

Prussian Blue Analogues-derived $ZnFe_2O_4$ in $CuO/ZnFe_2O_4$ p-n junction for H_2 production

Linh Trinh,[*,†] Aleksandra Parzuch,[†] Krzysztof Bienkowski,[†] Piotr Wróbel,[‡] Marcin Pisarek,[¶] Grzegorz Kaproń,[§] and Renata Solarska[*,†]

†*Laboratory of Molecular Research for Solar Energy Innovations, Centre of New Technologies, University of Warsaw, Stefana Banacha 2c, 02-097 Warsaw, Poland*

‡*Faculty of Physics, University of Warsaw, Ludwika Pasteura 5, 02-093 Warsaw, Poland*

¶*Institute of Physical Chemistry, Polish Academy of Sciences, Marcina Kasprzaka 44/52, 01-224 Warsaw, Poland*

§*Falculty of Geology, University of Warsaw, Zwirki i Wigury 93, 02-089 Warsaw, Poland*

E-mail: l.trinh@cent.uw.edu.pl; r.solarska@cent.uw.edu.pl

[C]S1

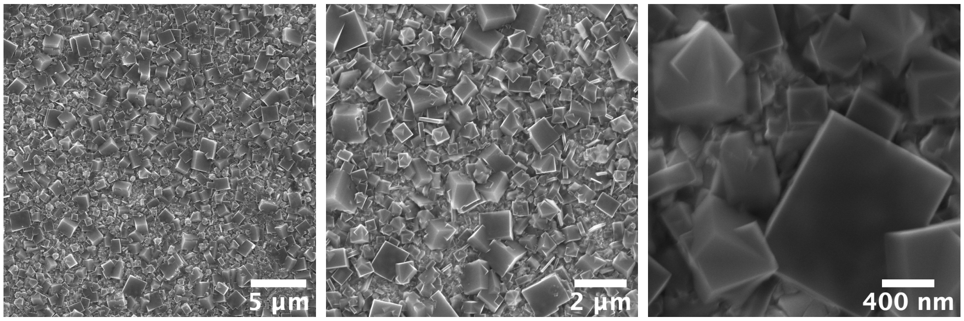

Figure S1: SEM of ZnHCF before annealing

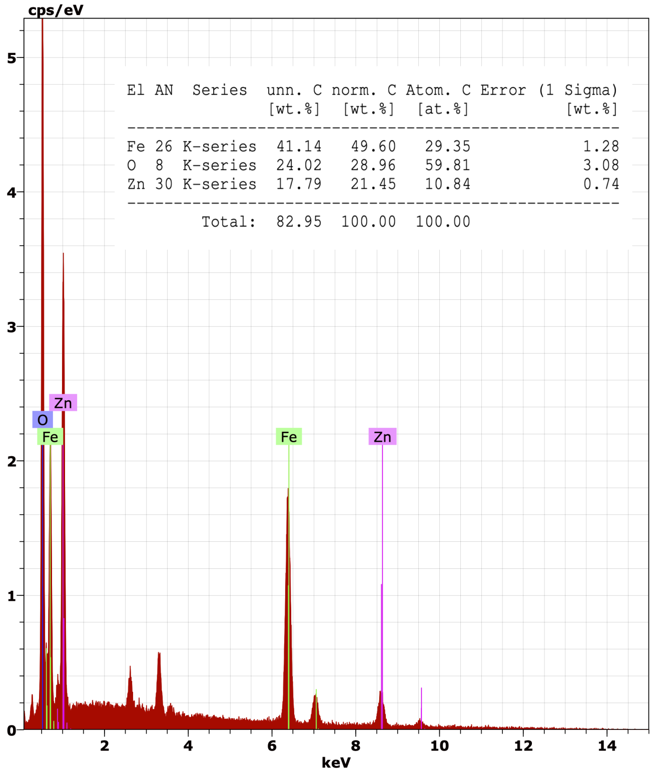

Figure S2: EDS analysis of $ZnFe_2O_4$ after annealing

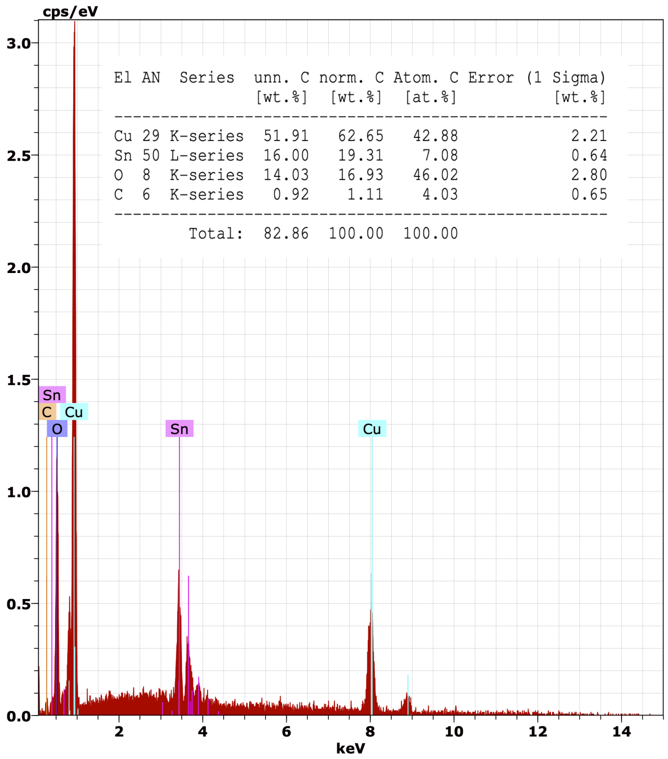

Figure S3: EDS analysis of CuO

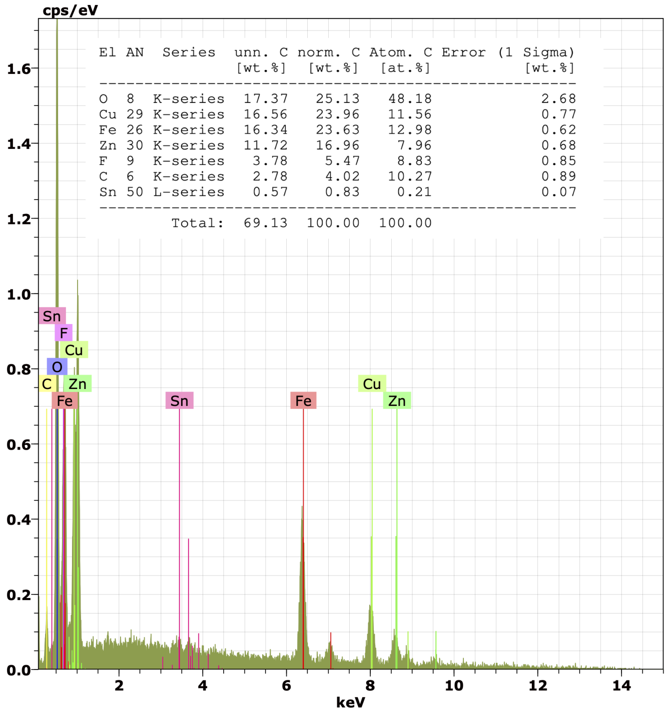

Figure S4: EDS analysis of $CuO/ZnFe_2O_4$

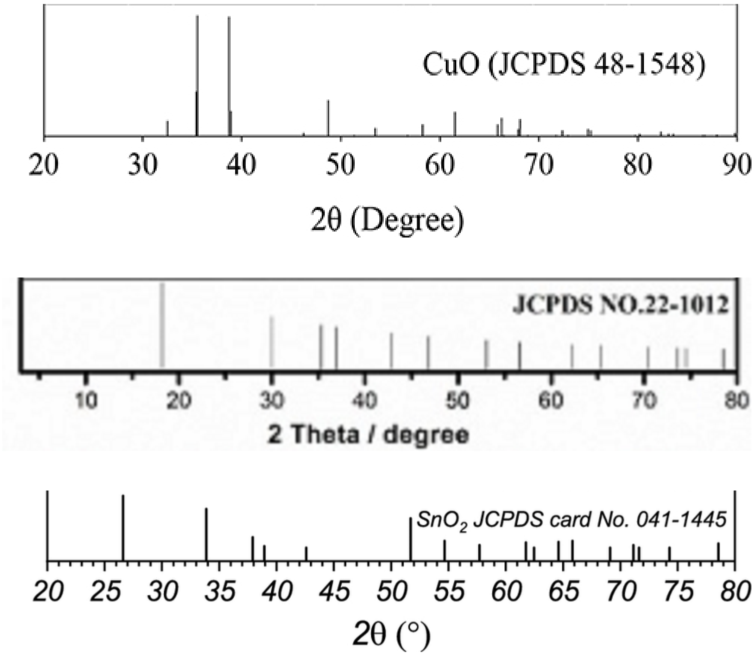

Figure S5: XRD patterns of standard CuO (JCPDS-48-1548), $ZnFe_2O_4$ (JCPDS 22-1012), and FTO (JCPDS 041-1445)

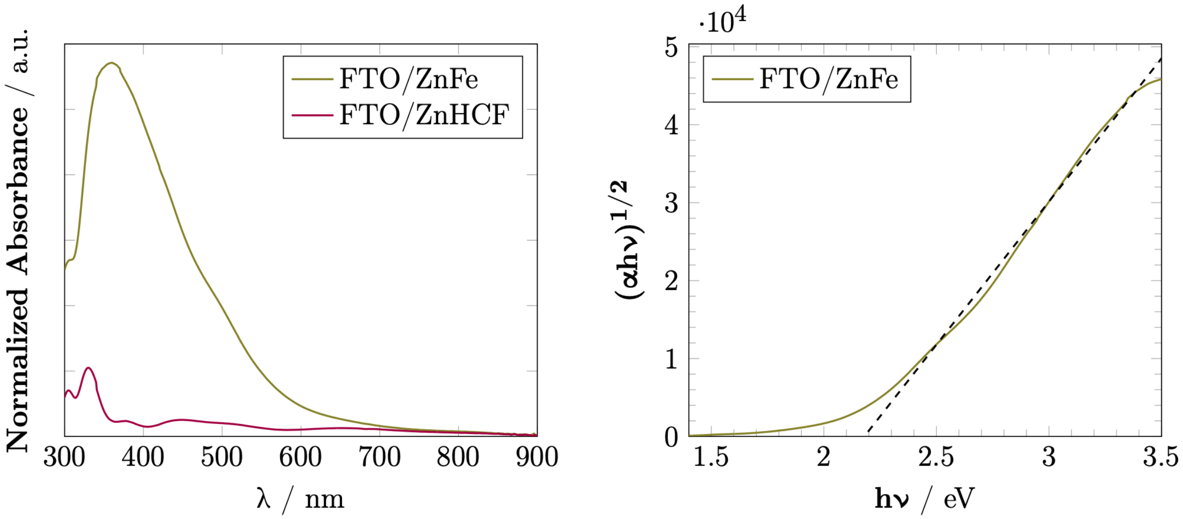

Figure S6: UV-Vis spectra of ZnHCF and $ZnFe_2O_4$ (left) and Tauc-plot of $ZnFe_2O_4$ (right)

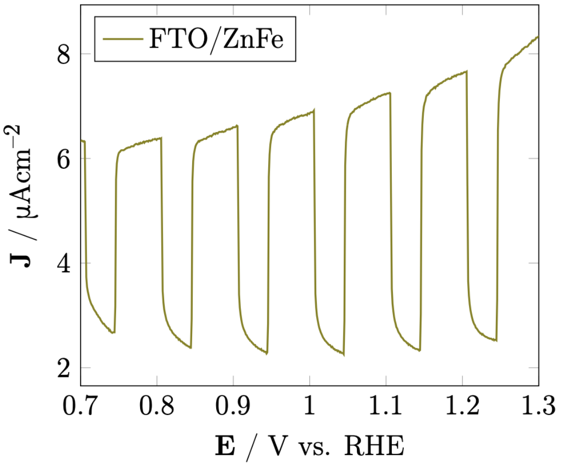

Figure S7: LSV of $ZnFe_2O_4$ in 0.5 M Na_2SO_4 (pH = 6.5)
